# Supplementary figures and images for: Evaluation of SARS-CoV-2 Seroprevalence and Variant Distribution During the Delta–Omicron Transmission Waves in Greater Accra, Ghana, 2021
Source: Viruses. 2025 Mar 28;17(4):487. doi: 10.3390/v17040487 (PMC12031444; doi:10.3390/v17040487)

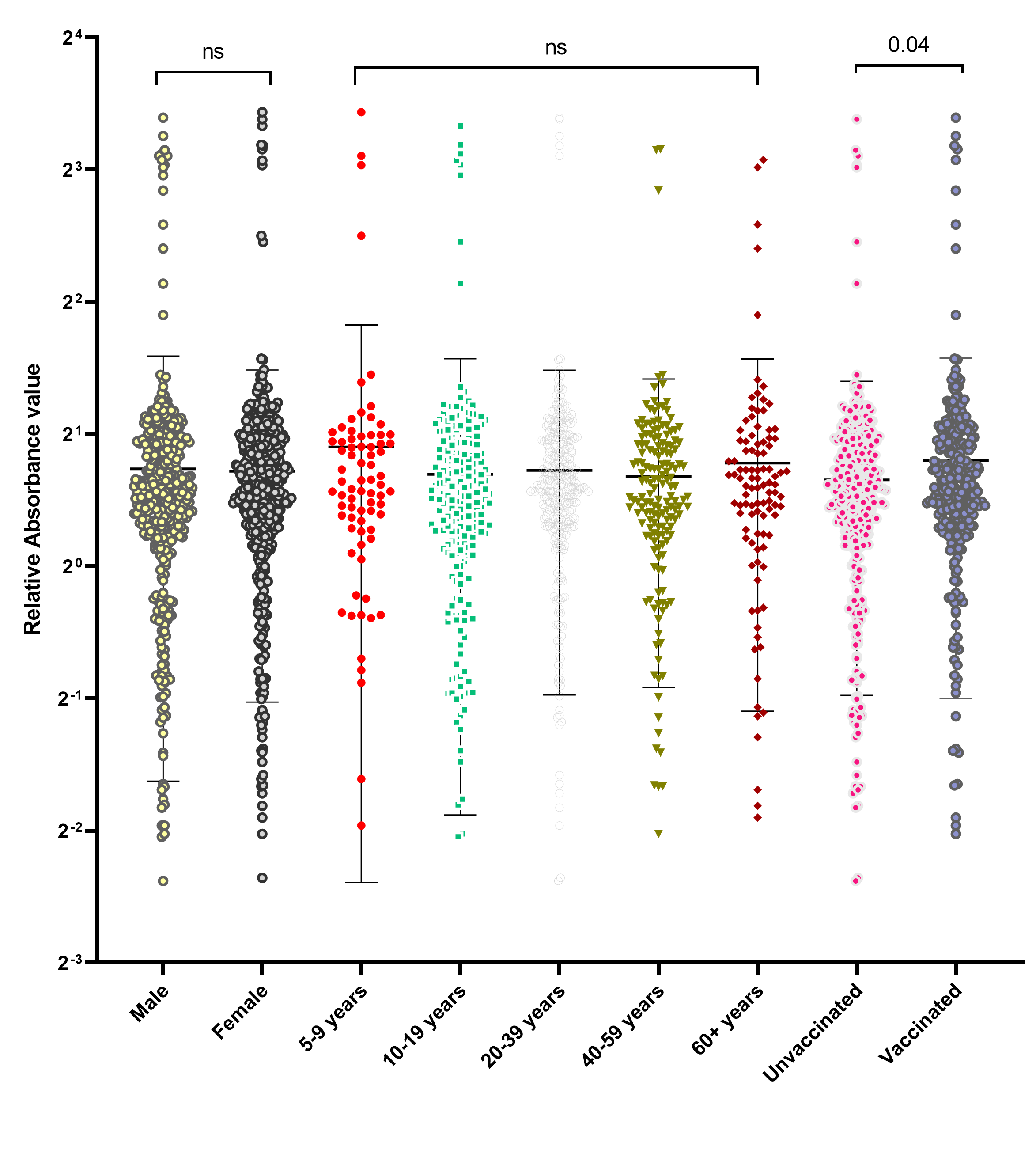

Supplement: Supplementary file 1 [file viruses-17-00487-s001.zip › viruses-3446867-supplementary.tif]
